# ADAPTATIONS AND OUTCOMES OF A WALKING AND REMINISCENCE BRAIN HEALTH INTERVENTION FOR CAREGIVERS

**DOI:** 10.1093/geroni/igae098.1269

**Published:** 2024-12-31

**Authors:** Patrice Fuller, Charles Fennell, Nora Mattek, Sarah Gothard, Jeffrey Kaye, Raina Croff

**Affiliations:** Oregon Health & Science University, Portland, Oregon, United States; Oregon Health & Science University, Portland, Oregon, United States; Oregon Health & Science University, Portland, Oregon, United States; Oregon Alzheimer’s Disease Research Center, Oregon Health & Science University, Portland, Oregon, United States; Oregon Health & Science University, Portland, Oregon, United States; Oregon Alzheimer’s Disease Research Center, Oregon Health & Science University, Portland, Oregon, United States

## Abstract

The SHARP-Caregiver (SHARP-CG) study evaluated the feasibility of adapting SHARP to family caregivers of care partners with mild cognitive impairment or early-stage dementia. SHARP-CG assigned 7 triads (n=21) to Group A or B. Triads had a family caregiver (age >40), their care partner (age >40), and a support person (age >18). Caregivers and support persons were healthy or mildly cognitively impaired. Triads walked 1-mile routes with images to prompt conversational reminiscence 3x a week for 16 weeks using the SHARP walking application. Caregivers and care partners contributed weekly health update data, and (optionally) sleep and step-count data. Group A participants walked immediately. Group B participants first completed 16-weeks of observation. Ages were 35-90 (mean 69.8); most were female (52%). Mean Montreal Cognitive Assessment score for caregivers and care partners was 21.7 (SD+4.6). Eighty-six percent (n=6) of caregivers and 71% of care partners (n=5) opted to engage in digital biomarker data collection (actigraphy watch and sleep sensor). Caregivers had greater mean total daily steps (2055; SD+686) than care partners (1684; SD+979). Mean sleep hours were similar for caregivers and care partners at 6.1 (SD+1.1) and 6.5 (SD+2.5). Recruiting caregivers was difficult because many family members did not recognize what they were doing as caregiving. To improve study accessibility for caregivers, adapted eligibility criteria included reducing minimum age, allowing mobility aids, and making some components optional. Increasing accessibility, while improving enrollment, did not necessarily impact adherence. Caregiver demands and sporadic health concerns limited participation.

